# Public Water Policy Knowledge and Policy Preferences in the American West

**DOI:** 10.3390/ijerph19052742

**Published:** 2022-02-26

**Authors:** Erika Allen Wolters, Brent S. Steel, Muhammed Usman Amin Siddiqi, Melissa Symmes

**Affiliations:** School of Public Policy, Oregon State University, 300 Bexell Hall, Corvallis 97331, OR, USA; erika.wolters@oregonstate.edu (E.A.W.); siddiqim@oregonstate.edu (M.U.A.S.); symmesml@oregonstate.edu (M.S.)

**Keywords:** environmental values, public agriculture knowledge, public water knowledge, water policy

## Abstract

The Western United States has made significant contributions to agricultural products both domestically and internationally. As the Western U.S. continues to grapple with water scarcity and extended periods of drought, evidence of misalignment between crop production and the volume of water necessary to maintain abundant food yields is becoming more pronounced. There are several policy nudges and mitigation strategies that can be employed to bring water availability and crop selection into alignment. Whether there is public support for these policies, or knowledge of how policies could impact water use in agriculture, it is important to understand what those preferences are and how people weigh tradeoffs between developing agricultural and water use. Using random household surveys of residents in the western U.S. states of Washington, Oregon, Idaho, and California, this study explores public water knowledge, the correlates of public water knowledge, and the impact knowledge has on preferred water policies while controlling for demographic characteristics, environmental efficacy, climate change belief, and political ideology. Findings show that knowledge does have an independent impact on preferred approaches to water policies while controlling for demographic characteristics, environmental efficacy, belief in climate change, and political ideology. Respondents who are knowledgeable about water recycling for food and water use for agriculture were significantly more supportive of water conservation policy approaches and less supportive of water supply-side approaches.

## 1. Introduction

In the summer of 2021, the first-ever water shortage was declared for the Colorado River, leaving Arizona farmers unable to irrigate their crops for the summer season [1]. In Oregon, the Klamath Basin drought resulted in an irrigation water shut-off, causing significant negative financial impacts to the agriculturally dependent region. After years of drought conditions, some of the most critical reservoirs in the American West, such as Lake Mead, are running low leading to temporary, but unsustainable, water transfers to mitigate the worst of the drought impacts [2]. The lack of water in the summer of 2021 was so significant that over 90 percent of the American West was deemed to be in a drought. Fall conditions did not impact the drought conditions in the West, with several regions in December 2021 still considered in “extreme” or “exceptional” drought [3]. The persistence of drought conditions suggests that this may be the new norm, and current “solutions” more likely focus on emergency reallocation of water instead of long-term solutions to mitigate water scarcity.

As many surface waters have been over-allocated and unable to meet demand, the reallocation of groundwater has been used either to supplement insufficient surface water or become the primary water source. This dependence on groundwater aquifers in the American West is causing substantial groundwater depletion and raising concerns about the ability of groundwater aquifers to recharge water supply fast enough for demand. Further, in the most extreme cases, over-pumping of groundwater from aquifers is causing land subsistence. In some regions of the Central Valley in California, land subsistence is occurring at more than 1 foot per year [4], which in the worst case could result in the inability of the aquifer to act as a future water bank for freshwater.

As a finite resource, there are limited options to mitigate water scarcity. Large-scale infrastructural dam and pipeline projects are becoming an anachronism of an era when water was considered abundant and necessary for the expansion and development of the West. The remnants of these projects are currently evident in the misalignment between crop production and the volume of water necessary to maintain abundant food yields, water shut offs in agricultural regions, as well as more prevalent water restrictions in urban areas. A greater push toward water conservation is apparent with more households planting xeriscaped yards, ubiquitous household installations and purchasing of water-efficient appliances and devices, and agricultural producers utilizing more water-efficient irrigation and growing less water-intensive crops [2]. Additionally, technological innovations have expanded the definition of “fresh” water with desalination plants converting saltwater to potable water and more recycled water being employed not only for irrigation but other residential and industrial uses. 

Residents in the West are keenly aware of water scarcity issues. A 2019 poll by Colorado College found that a majority of residents in the American West had high levels of concern for water scarcity [5]. The Pew Research Center found that 76% of residents in the Pacific states recognized the impacts of climate change on periods of “drought or water shortages” [6]. Further, recent water restrictions to several Western cities and other regions have helped to create an awareness of water scarcity, potentially making water policy salient to the public. Using random household surveys of residents in Washington, Oregon, Idaho, and California, this study explores public water knowledge, the correlates of public water knowledge, and the impact knowledge has on preferred water policies. 

Awareness of water scarcity and drought may raise concern over water management and conservation, potentially leading to greater water knowledge. While other studies have examined the impact on knowledge on conservation policies in specific regions, few explore the publics’ knowledge and related water policy preferences on geographically diverse regions. Using random household surveys of residents in Washington, Oregon, Idaho, and California, this study explores public water knowledge, the correlates of public water knowledge, and the impact knowledge has on preferred water policies. If water knowledge correlates to preferred water policies, then a clearer pathway for policymakers to garner public support for water conservation may emerge.

## 2. Literature Review

### 2.1. Background

Traditional freshwater sources are drastically suffering from climate change and overuse [7], with both population growth and climate variability significantly impacting water availability around the world [8]. Around 70% of the world’s freshwater is allocated for irrigation [9]. On average, in the United States, 80% of the total water consumption per year is associated with agricultural use [9], with agriculture in the Western states accounting for 90% of total water consumption [9]. Historical water use in the region spurred the development of Western cities but was also the foundation of the development of the agricultural industry, with the region now providing a significant amount of agricultural products both domestically and internationally. Securing a dependable water supply for agriculture and irrigation is crucial for both crop production and the economies of the West, but also to minimize impacts of water scarcity on other water uses (e.g., ecological, industrial, residential, recreational, etc.).

Even though recycled water benefits the environment, economy, industry, and agricultural communities, it is still considered a controversial topic with public acceptance of recycled water dropping “with increasing human contact” [10]. Thus, many industries that could use recycled water do not at this time due to lack of acceptance or availability [7]. Negative perceptions reflect human health concerns and food safety [7], as well as the “yuck” factor [11] with people imagining sewage water turned to drinking water or other potable uses, thus finding it an unsavory conservation option. Even during periods of drought, public acceptance of recycled water remained unaffected [12], suggesting that there is a significant lack of knowledge about the safety and cleanliness of recycled water.

### 2.2. Water Profiles of California, Idaho, Oregon, and Washington

The American West produces a significant amount of the nation’s agricultural products, requiring vast sums of water to meet irrigation needs. In 2015, total water withdrawal for irrigation purposes amounted to approximately 74% of California’s water withdrawal, 86% of Idaho’s, 78% of Oregon’s, and 59% of Washington’s with each state utilizing a significant, if not majority, of both surface and groundwater withdrawals for irrigation [13]. Water use contributes directly to all four states’ agricultural production and economies. In 2019, estimated agricultural revenue in California was USD 49.9 billion, USD 8 billion in Idaho, USD 5 billion in Oregon, and USD 9.3 billion in Washington [14]. Washington, Oregon, and Idaho collectively are the top producer of 22 principal agricultural products [15], while California produces over one-third of the nation’s vegetables and two-thirds of its fruits and nuts [16]. While increasing water scarcity threatens crop management and abundance, water efficiency for agriculture has not necessarily led to less water use, in some cases increasing the crop size since more water is available [17]. With the significance of irrigated crops in CA, WA, OR, and ID, on both food production and regional economies, addressing water use and management is critical to maintaining economic and production stability.

### 2.3. Public Knowledge and Water Policies

Electing delegates to act on behalf of citizens in policymaking institutions does not mark the end of citizen participation in representative democracies. The ideal of democratic governance demands that citizens should be able to actively speak out on government actions and effectively steer policy development [18,19]. However, when it comes to complex policy areas such as water resource management, scholars have been especially concerned about the democracy and technical information quandary; that is, how does one legitimately push for public involvement in policy areas where the level of public awareness is not commensurate with the complexity of the respective issues [20,21]. The impression that the public does not always have adequate knowledge about policy issues has led some to even question the value of public participation in policymaking. From the Platonic notion of ‘philosopher king’ to Lippmann’s ‘phantom public’ in the early 20th century, the calls for the rule of experts have stemmed from the concern that the public generally lacks policy-relevant knowledge [22]. Even in the 21st century, some well-known climate researchers and environmentalists, frustrated by the lack of action, have endorsed authoritarianism as an answer to climate change [23,24]. However, as Stehr puts it, what we really need is more democracy but with enhanced “knowledgeability of individuals, groups, and movements who work on environmental issues” [25] (p. 44).

Citizens’ policy preferences are intendedly rational; however, their rationality is bounded by the knowledge they possess about respective policy issues [26,27,28,29,30]. Consequently, fallacious knowledge claims are likely to culminate in a choice of unwarranted solutions to problems [31,32], especially in dealing with complex problems where individuals often heavily rely on heuristics [33]. Adequate policy-relevant knowledge is vital for citizens to not only assess policy alternatives but also to enable them to discern their real interests and influence policymaking accordingly [34,35,36,37]; as Janicke argues, “without knowledge, there is no (perceived) problem, no public awareness, and consequently no policy process…” [38] (p. 7). At the policymaking level, policy process theories incorporate the vital role of knowledge by assuming boundedly rational policymakers who are bounded by beliefs in decision making [39,40] and by time and attention to agenda setting [41,42].

Prior research has identified a knowledge gap between experts and the public [43,44,45], which can culminate in bad policies, as studies show that when public views diverge from that of the experts, policymakers tend to go along with popular opinion [46,47]. This knowledge gap dovetailed with Americans’ ideologically charged and divergent views about the existence and severity of climate change [48] is what motivates our study. Given that most scientists argue that human-caused greenhouse gas emissions are the main source of climate change and that climate change is leading to increased drought and water shortages in many areas of the world, especially in the U.S. west, it is important to assess the scope of public knowledge concerning water resources and to identify the link between knowledge and support for various water management policies. By more clearly specifying the connection between knowledge levels and the impact—if any—on beliefs about water policy preferences, one can better understand how information dissemination efforts may be designed to more effectively engage the public in water management issues and to assist the public in understanding policy discussions that concern water. Science communication experiments in the U.S., Canada, Europe, Asia, and Africa have shown encouraging findings that individuals’ policy preferences are likely to change when they are equipped with relevant knowledge through effective communication and public deliberations [49,50,51,52,53,54].

Earlier studies indicate that water literacy among the U.S. population is generally at a very low level. The majority of the citizens have not been found well informed about water-related terms or issues concerning water resources management [55,56,57,58,59]. McCarroll and Hamann have recently reviewed two streams of studies related to water literacy, one focusing on students and the other on adults (18 years and above). They have found that knowledge gaps and misconceptions related to water resources among the students are carried through to adulthood [60]. Studies in educational research have shown that students often struggle to understand water-related concepts [61,62,63,64], and the trend persists in the general public that exhibits a limited understanding of water-related policy issues [55,56,57,58,59,60,65,66].

Several studies on the correlates of policy-relevant knowledge have identified a knowledge gap between people of higher and lower socioeconomic status (SES) [36,37,67]. Individuals with higher SES have not only been found to exhibit higher levels of knowledge holding but also tend to more quickly respond to efforts to increase public knowledge compared with those with lower SES [68,69]. Education and income are typically found to be positively associated with water literacy [70,71] and public knowledge related to policy issues in general [37,67,72,73,74,75]. However, a recent study of water literacy among the urban residents of China reveals a negative relationship between income and water-related knowledge [71].

While age is generally found to be positively related to knowledge holding [37,76], younger people were found to be more knowledgeable than older cohorts in the case of water-related knowledge [32,33,72,73]. However, Dean et al., found age to be positively related to water literacy [70]. Several studies found male respondents to be better informed than females about policy-related issues in general [37,72,75,76,77] as well as about issues specifically related to water resources [70,78]. However, female respondents were found to be more knowledgeable in several policy issues than males in Turkey, Palestine, and Taiwan [34,35,36,73,79,80].

Political ideology, climate change beliefs, and perceptions of self-efficacy are also included in this study as correlates of water literacy and policy support because knowledge is not just influenced by demographics and socioeconomic status but also by values, perceptions, personal interests, and life experiences that contribute to associative learning [34,74,75,81,82,83,84,85]. Research shows that liberals are significantly different in their orientation toward science than those who identify themselves as conservatives—the former believing scientists and science to be objective [86]. Liberals, therefore, have a higher level of motivation to acquire scientific knowledge, and as a corollary, are more likely to exhibit a higher level of knowledge in complex policy domains such as water resource management.

Research exploring variables affecting acceptance of recycled water illustrates variation among demographic groups. Regarding age, older consumers are less likely to accept the uses of recycled water than younger ones [9,12]. The intuition behind these findings is that older people are more concerned with health risks given their increased health concerns as older adults [9]. Conversely, research has revealed that educated and environmentally conscious people are more willing to use and accept recycled water [87]. Gender has also been found important with men more likely to support recycled water use compared with women [10,12]. In a meta-study conducted by Fielding, Dolnicar, and Schultz (2019), higher income levels were consistently correlated with higher levels of acceptance of recycled water [10]. Cost is also significant in encouraging the usage of recycled water [87]; more specifically, the price should reflect the price of freshwater used for irrigation during water scarcity, and not cost more [87]. Further, a study by Glick et al. (2019) examined policy-relevant knowledge and support for recycled water, finding a correlation between increased knowledge and support for recycled water use [12].

Acceptance of water conservation strategies, such as using recycled water, might only gain greater public acceptance if there are higher levels of knowledge pertaining to current water use in agriculture. A recent national survey of U.S. adults found that a majority (53%) were knowledgeable of the connectivity between water and food production [88], recognizing the trade-offs and water concerns in agricultural food production. While the U.S. public has some water knowledge pertaining to agricultural use and concern about abundance, there is potentially a lack of knowledge pertaining to water resources themselves. A 2018 study of Texas residents found that a vast majority (between 79% and 95% of mail and online respondents, respectively) expressed the belief that groundwater is an abundant resource that “is plentiful and will always be available for human use” [89]. Understanding both the amount of water necessary for agricultural production as well as the source of irrigation water is therefore critical in terms of public preferences of water policy.

Prior research suggests that generally there is low water knowledge and literacy among the public. Policymakers often respond to public opinion regarding policy preferences suggesting that potentially some water conservation policies that are needed would not be preferred by the public. This research adds to the growing literature examining the correlates of the public’s water knowledge as it relates to policy preferences. If, as prior studies suggest, there is a knowledge gap or water literacy is indeed low, how might this impact policy preferences? In other words, is water knowledge and literacy a significant factor in support for water conservation policies? Results from this study will provide greater insight into how public water policy preferences can inform policymakers or suggest ways in which public support for water conservation policies could potentially be enhanced through targeted public education efforts.

## 3. Methods

### 3.1. Research Questions

The study aims to investigate two research questions: (a) how water literacy is associated with demographic characteristics and value orientations of citizens in the American West; and (b) whether water literacy affects public support for water policies pertaining to supply-side and demand-side (conservation) approaches after controlling for demographic characteristics and value orientations?

### 3.2. Sample

The study is based on data obtained from a survey of 1804 respondents randomly selected from four Western states: California, Idaho, Oregon, and Washington. An initial sample of 4695 households from all four states was randomly selected based on addressed-based sampling (ABS) in 2018. The sample households with valid residential addresses were selected by a national sampling company using the U.S. Postal Service’s Computerized Delivery Sequence (CDS) file. Each of the above-mentioned states had almost equal representation in the sample, ranging from 1170 to 1177 households. An adult member of the household (18 years or above) who had most recently celebrated a birthday was asked to complete the survey.

### 3.3. Survey

The survey was administered following a modified version of Dillman’s Tailored Design Method (TDM) [90]. Grounded in social exchange theory that explains individuals’ motivation to engage in certain social behaviors, the TDM aims at simultaneously improving the response rate and mitigating the sources of survey error. Capitalizing on human perceptions of costs, benefits, and trust, the TDM proposes techniques to design the questionnaire as well as the procedures for survey implementation that can enhance respondents’ trust that the potential benefits of responding take precedence over the costs of responding. A postcard was sent out to all households in our initial sample that notified them of the survey and provided them with an option to complete the survey online. Those who did not choose to opt out or complete the online survey received a mail-in survey in the second wave. The third wave of mail-in surveys, accompanied by a reminder letter, was sent to households that had not responded or opted out of participation. A pre-paid, first-class return mail envelope was enclosed with all the mailings to reduce the cost of responding. All the respondents were informed that their participation in the survey is completely voluntary and were asked to give their consent upon completion and submission of the survey. The respondents were apprised of the purpose and scope of the project through an accompanying cover letter, which was hand signed by the principal investigator (PI) and provided the contact information of the PI for respondents to reach out for queries. Survey responses are stored on password and virus-protected computers to ensure the privacy of the respondents. The study was designed to meet ethical standards of human subject research and was approved by the Institutional Research Board (IRB) at Oregon State University (OSU) on 30 November 2017 (study number: IRB #8349). The project was supervised by OSU faculty, and everyone involved in the project was certified to conduct human subjects research after training.

### 3.4. Case Studies

**Figure 1:**
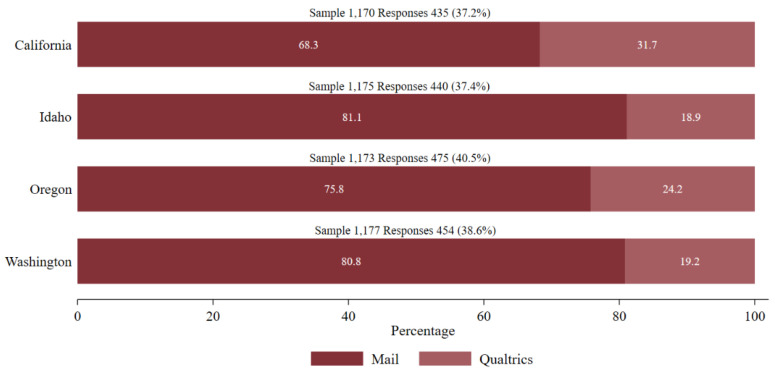
State-wise Response Rates.

California, Idaho, Oregon, and Washington were selected as case studies based on similarities in their ecosystem services and policymaking institutions as well as a shared historic context of water shortages and drought. All these states are experiencing ongoing water shortage issues generating state-wide debates about suitable water policies. Figure 1 shows that response rates from the four states were fairly similar, ranging from 37.2% (CA) to 40.5% (OR), with an overall response rate of 38%. In general, most of the respondents opted for the mail-in survey. As a percentage of the completed surveys from each state, the online completed surveys ranged from 18.9% (ID) to 31.7% (CA). Table 1 compares the distribution of the respondents from the four states based on age, gender, education, and income with that of the population of their respective states according to 2010 Census data. The sample is generally representative of the populations in the four states but is slightly older and has higher levels of formal education and income. However, this trend is consistent with survey research.

### 3.5. Operationalization and Descriptive Statistics

*Dependent variables:* We have a total of 10 dependent variables: two variables related to public water knowledge and six related to support for different water policies. Public water knowledge was assessed using a quiz on the food–water–energy nexus developed by Portney et al. [91], which asked respondents if certain statements were “accurate”, “inaccurate”, or “do not know”. The statements concerning water were: “Recycled water cannot be safely used to grow food,” and “Crop irrigation in the U.S. uses more groundwater than all other uses combined.” Given the discussion above, the correct answer for the first statement is *inaccurate* and for the second statement, it is *accurate*. The recycled water and food statement was dichotomized so that 1 = inaccurate (correct answer), 0 = else, and for the crop irrigation question 1 = accurate (correct response), 0 = else. The survey included a variety of possible water management policies developed by Portney et al. [91]. Respondents were asked about their level of opposition or support on a Likert-type scale ranging from 1 = strongly oppose to 5 = strongly support. Policy support variables were dichotomized so that 1 = support (support or strongly support), 0 = else (neutral, oppose, or strongly oppose). Descriptive statistics of the dependent variables are presented in the analyses section.

*Independent and Control Variables:* The independent variables included in the analysis are presented in Table 2 below. Age in years, a gender dummy variable, formal educational attainment and household income are included for demographic variables. The environmental efficacy index was developed by adding individual scores on the following four statements with a five-point response scale ranging from 1 = “Strongly Disagree” to 5 = “Strongly Agree”: “I feel that my own personal behavior can bring about positive environmental change”; “I would be willing to accept cuts in my standard of living, if it helped the environment”; “I would be willing to support higher taxes, if it helped to protect the environment”; and “I would be willing to sacrifice some personal comforts in order to conserve resources.” The composite efficacy scale ranged from 4 = low efficacy to 20 = high efficacy [92]. The climate change beliefs variable was determined based on the question: “From what you’ve heard or read, do scientists generally agree that the Earth is getting warmer because of human activity, or do they not generally agree about this?” Responses were collapsed and recoded into dichotomous categories where 1 = Yes, the Earth is getting warmer because of human activity and 0 = else (those who answered do not know or not due to human activity). Political ideology was measured using a nine-point scale with 1 = “Very Liberal” to 9 = “Very Conservative”. Mean results on political ideology show a moderate population with a slight liberal leaning (mean = 4.68).

### 3.6. Analytic Approach

We used a combination of descriptive and analytical statistics to address our research questions of interest. The logistic regression technique was used for multivariate analyses as all of our dependent variables were operationalized in binary form. Table 3 and Table 4 present frequencies of correct responses to our water knowledge questions. Logistic regression models predicting public water knowledge included demographic characteristics (age, gender, education, income, and state) and value orientations (environmental efficacy, climate change views, and political ideology) as independent variables. For the second research question, we first used t-tests to compare policy support mean scores between respondents with correct and incorrect responses for the two knowledge questions, which confirms our research proposition that those with knowledge of water resources would be more supportive of water conservation policies and less supportive of supply-side approach policies. Finally, to assess the effect of water knowledge indicators on policy support dependent variables, we used logistic regression models while controlling for the demographic characteristics and value orientations.

## 4. Analyses

Table 3 and Table 4 present frequencies of responses to the following water knowledge statements by state: (a) “Recycled water cannot be safely used to grow food,” and (b) “Crop irrigation in the U.S. uses more groundwater than all other uses combined.” Overall, an overwhelming majority of respondents in each state identified the first statement correctly as inaccurate with Oregon having the highest percentage at 83.1 percent and California with the lowest percentage at 74.4%. California respondents were in the highest percentage to respond that the statement was accurate at 12.0 percent, while for Washington respondents, only 4.0 percent identified the statement as accurate (i.e., incorrect answer). Washington had the highest percentage of respondents responding “do not know” at 20.4 percent while the other three states ranged from 10.8 percent in Oregon to 13.6 percent in California. The chi-square statistic was also significant at the 0.000 level, indicating that the differences between the states are statistically significant.

Table 4 displays frequencies by state for the second statement that crop irrigation in the U.S. uses more groundwater than all other uses combined. When compared with the first statement concerning recycled water, fewer respondents had the correct response (i.e., accurate). Idaho had the largest percentage of respondents with the correct answer at 37.7 percent, followed by Oregon at 34.3 percent, Washington at 31.4 percent, and lastly, California at 24.3 percent. California had the highest percentage of respondents with an incorrect response at 26.7 percent and Washington had the lowest percentage with a wrong answer at 10.1 percent. The most prevalent response in each state was “do not know,” with a range of 58.5 percent for Washington respondents and Idaho with the lowest percentage among the four states at 39.1 percent.

Logistic regression estimates are provided in Table 5 for the effect of the demographic and value orientation variables as well as state dummies on the responses to the two water statements. The results are presented in odd ratios and their confidence intervals. The chi-square statistics for both logistic regression models are significant, meaning that both models are a good fit, statistically speaking. The percent of cases correctly classified for the recycled water model is 80.1 percent, and the pseudo R^2^s are 0.089 (Cox and Snell R^2^) and 0.137 (Nagelkerke R^2^). For the crop irrigation model, the percent correctly classified is 68.2 percent, and the pseudo R^2^s are 0.061 (Cox and Snell R^2^) and 0.104 (Nagelkerke R^2^). These are not large coefficients of determination for either model, but this is typical for public opinion research. For the first model concerning recycled water, the only statistically significant result for the demographic variables was education. Respondents with higher levels of education were significantly more likely than lower educated respondents to respond that the statement “recycled water cannot be safely used to grow food” is inaccurate. For the crop irrigation and groundwater model, all four demographic variables are significant. Younger, male, more highly educated, and higher-income respondents were significantly more likely to respond with the correct answer than older, female, lower educated, and lower-income respondents.

Turning now to the value orientation variables, we find that the environmental efficacy and climate change beliefs were statistically significant for both recycled water and crop irrigation models. Those respondents with higher levels of environmental efficacy are significantly more likely than those with lower levels of efficacy to respond that the recycled water and food statement was inaccurate (correct answer) and the crop irrigation statement was accurate (correct answer). Similarly, those respondents who believe the Earth is getting warmer due to human-caused activities were 86 percent more likely than those who do not believe in human-caused climate change to respond that the recycled water and food statement is inaccurate, and 42 percent more likely to respond that the crop irrigation statement was accurate. However, political ideology was not significant in any of the models.

In summary, the overwhelming majority of respondents in each of the four states responded that the statement “recycled water cannot be safely used to grow food” is “inaccurate”. However, respondents from Idaho and Oregon, respectively, were 61 and 74 percent more likely to respond with the correct answer than respondents from California. Respondents from Washington were not statistically different from Californians. Those who responded that it is an “accurate” statement or “do not know” tended to have lower levels of education, lower levels of environmental efficacy, and do not believe in human-caused climate change. While far fewer correctly responded that “crop irrigation in the U.S. uses more groundwater than all other uses combined”, older, females, those with lower levels of education, lower household incomes, and lower levels of environmental efficacy were more likely to respond that the statement was inaccurate or stated they “do not know”. Respondents from Idaho, Oregon, and Washington, respectively, were 2.46, 1.76, and 1.44 times more likely to identify the correct answer for the crop irrigation statement.

### Policy Implications of Water Knowledge

The next set of analyses investigates the role that water knowledge has on water policy preferences. The survey included a variety of possible water management policies developed by Portney et al. [91]. Six of the water policies are provided in Table 6 and Table 7 controlling for the two water knowledge statements. Respondents were asked their level of opposition or support on a Likert scale ranging from 1 = strongly oppose to 5 = strongly support. Mean scores are displayed for those that responded with the correct answer compared with those who responded with the wrong answer or said they “do not know”. We expect that those respondents with correct answers for both water knowledge statements to be more supportive of water policies that focus more on conservation efforts (demand side) and less supportive of policies that promote increasing water supply when compared with those with a wrong answer or “do not know”. It is likely that those with correct answers are more nuanced with the current state of water supply and use in the western U.S., and more likely to consider the future of water with climate change effects.

The results displayed in Table 6 reveal that those respondents with the correct answer to the statement “recycled water cannot be safely used to grow food” were significantly less supportive of “build dams and reservoirs” and “build pipelines to bring water from other regions” than those with wrong answers or who said “do not know.” While the significance level of the t-test for “build dams and reservoirs” is at the 0.01 level compared with the 0.001 level for the “build pipelines” policy, both were nonetheless significant. These were the only two policy statements that focus on water supply and not conservation. The next four policy statements focus more on conservation efforts of existing water supplies. Those respondents with correct responses for the “recycled water cannot be safely used to grow food” statement were significantly more likely to support the following policies when compared with those with wrong responses or who said “do not know”: “conduct campaigns for voluntary water conservation,” “give tax incentives for the installation of water-saving equipment,” “require low water use landscaping,” and “give tax incentives for implementing efficient irrigation systems for agriculture.” All t-test statistics were significant at the 0.001 level.

Table 7 reports mean scores for the same six water policies controlling for answers to the statement “crop irrigation in the U.S. uses more groundwater than all other uses combined.” Similar to the results presented in Table 6, those respondents with incorrect or “do not know” responses to the statement were significantly more likely than those with correct responses to support “build dams and reservoirs” and “build pipelines to bring water from other regions.” Once again, these are both supply-side policies to increase existing water resources. For the remaining policies that promote conservation, there are statistically significant differences in levels of support for all four policies at the 0.001 level. Those respondents that correctly identified the statement as accurate are significantly more likely to support “conduct campaigns for voluntary water conservation,” “give tax incentives for the installation of water-saving equipment,” “require low water use landscaping”, and “give tax incentives for implementing efficient irrigation systems for agriculture”.

The analyses presented in Table 6 and Table 7 confirm our research proposition that those with knowledge of water resources would be more supportive of water conservation efforts and less supportive of supply-side approach policies. However, a final set of multivariate analyses was conducted to control for the demographic characteristics, efficacy, and values previously included in the models with the two water knowledge indicators as dependent variables. Table 8 presents logistic regression models with demographic characteristics, value orientations, and state dummies included as predictors of water policy preferences. In addition, the two water knowledge indicators are also included in each model as independent variables. The chi-square statistic is statistically significant at the 0.001 level for each of the six models. The percent correctly classified ranges from 64.0 percent for the policy A model (build dams and reservoirs) to a high of 84.7 percent for the policy F model (give tax incentives for implementing efficient irrigation systems for agriculture). For the two pseudo R^2^ measures included for each model, the lowest coefficients were for the policy B model (build pipelines to bring water from other regions) with a Cox and Snell R^2^ of 0.079 and Nagelkerke R^2^ of 0.135. However, the pseudo R^2^ coefficients for the remaining models are much higher ranging from a 0.121 Cox and Snell R^2^ and a 0.206 Nagelkerke R^2^ for the policy A model to a 0.341 Cox and Snell R^2^ and a 0.476 Nagelkerke R^2^ for the policy C model (conduct campaigns for voluntary water conservation).

For the demographic variables in the six models, age had a significant and positive effect for Models A and E. Older respondents were more supportive of building dams and reservoirs and requiring low water use landscaping than younger respondents. Gender had a statistically significant effect in all models, with females less supportive of the supply-side policies of building dams and reservoirs and building pipelines to bring water from other regions when compared with males. Females were significantly more supportive of the three conservation-oriented policies (i.e., campaigns for voluntary water conservation and tax incentives for water-saving equipment as well as for efficient irrigation systems for agriculture) and less supportive of one conservation policy (requiring low water landscaping) than male respondents.

Education had a statistically significant effect in three models with the more highly educated more supportive of building dams and reservoirs, more supportive of voluntary campaigns for water conservation, and more supportive of providing tax incentives for installing water-saving equipment when compared with those respondents with lower levels of formal education. Income was significant for four models. Those respondents in higher income households are more supportive than lower-income households to support building pipelines to bring water from other areas, to provide tax incentives for the installation of water-saving equipment, and to provide tax incentives for efficient irrigation systems. Higher-income households were less supportive of requiring low water use landscaping. Higher income respondents are supportive of tax incentive approaches to water policy and least supportive of regulatory approaches.

Next, we examine the environmental efficacy, climate change belief, and political ideology variables. Environmental efficacy had a significant effect in all six models similar to gender. Those respondents with higher levels of efficacy are less supportive of the supply-side policies of building dams and reservoirs and building pipelines to bring water from other regions when compared with those with lower levels of efficacy. However, those respondents with higher levels of efficacy are significantly more supportive of all four conservation-oriented policies than those with lower levels of efficacy (policies C, D, E, and F). Climate change belief is significant in four of the six models. Those respondents who believe the Earth is getting warmer due to human-caused activities were less supportive of building dams and reservoirs (supply-side approach), and more supportive of all water conservation policies. Finally, ideology had a significant effect in four of the models. When compared with more liberal respondents, conservatives are significantly more supportive of building dams and reservoirs, significantly less supportive of the conservation policies of tax incentives for water-saving equipment requiring low water use landscaping, and tax incentives for efficient irrigation systems.

The water knowledge-related variables in the six models are the main focus of this study and concern the impact of knowledge on water policy options while controlling for demographic and value-orientation characteristics. The bivariate results displayed in Table 6 and Table 7 are prevalent in the multivariate analyses with two exceptions. The significance and direction of the coefficients are what was expected with the exception of one model: recycled Water was not significant for one model (i.e., policy A: build dams and reservoirs model). However, for those respondents who answered correctly that recycled water can be used safely to grow food, they are significantly less supportive of building pipelines, and significantly more supportive of the three water conservation policies including tax incentives for both water-saving equipment and efficient irrigation systems for agriculture, and requiring low water use landscaping. The crop irrigation variable had a significant effect in all six models in the direction that was expected. Those respondents who responded correctly to the statement that crop irrigation in the U.S. uses more groundwater than all other uses combined, were less supportive of the two supply-side water policies of building dams and pipelines and more supportive of the remaining four water conservation-oriented policies either through tax incentives, requiring low water use landscaping, or conducting voluntary water conservation campaigns.

**Figure 2:**
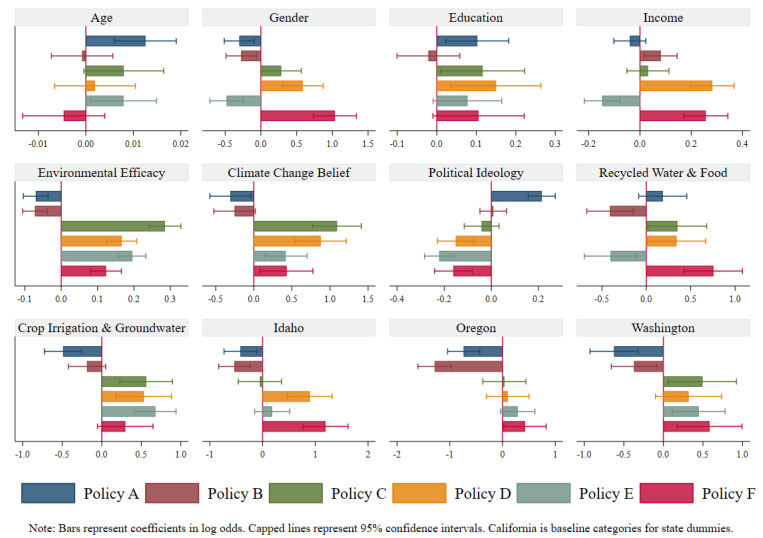
Policy Support Coefficients from Six Models for each Independent Variable.

Respondents from Idaho, Oregon, and Washington were significantly less supportive of both the supply-side policies (policy A and policy B) compared with respondents from California. Likewise, when compared with respondents from California, respondents from Washington were significantly more supportive of all water conservation policies, and respondents from Idaho and Oregon were more likely to support two and one conservation policies, respectively. These findings are in line with our research proposition as Californians were less likely to provide correct responses to water knowledge statements. For a clear picture of the effects of independent variables on policy support, Figure 2 presents independent-variables-wise coefficient plots of our six models.

## 5. Discussion and Conclusions

This study examined whether public water knowledge affects support for water policies pertaining to supply-side approaches (building dams and pipelines) and conservation policies (e.g., tax incentives for water efficiency). While earlier research indicates that the U.S. population is generally not well informed about water-related terms or issues concerning water resources management, our survey shows mixed findings about water literacy. The residents of the four states surveyed (CA, ID, WA, and OR) showed high levels of knowledge regarding the safety of recycled water for irrigation but much lower levels of knowledge pertaining to water use by irrigators. Multivariate analysis of water knowledge points to a knowledge gap between people of higher and lower socioeconomic status, which is in line with extant research on the correlates of policy-relevant knowledge in general [36,37,67,72,73,74,75] and that of water literacy in particular [71,72]. Of those respondents, those who had higher levels of education, a higher degree of personal efficacy, belief in anthropogenic climate change, and who identified as more politically liberal, were more likely to correctly select that it is inaccurate that recycled water cannot safely be used to grow food. Demographic characteristics and value orientations that were significantly correlated with accurate responses pertaining to irrigation using more groundwater than all other uses combined included younger people, males, people with higher levels of formal education, people with higher incomes, and those with a stronger sense of personal efficacy.

Demographic variables and value orientations were also found to relate to policy preferences to varying degrees. Age, gender, income, and education impacted policy support with older people preferring building dams and pipelines and requiring low water use landscaping, females more likely to support all four conservation policies, people with higher levels of formal education preferring building dams and reservoirs, voluntary efforts, and tax incentives, and those with higher incomes more supportive of building pipelines and providing tax incentives and less supportive of regulatory policies. More consistent predictors of water policy preferences were value orientations. People with higher levels of personal efficacy, belief in anthropogenic climate change, and who are more politically liberal support all four conservation policies and are less supportive of building dams and reservoirs or more pipelines.

Central to our research was to explore whether accurate responses to water knowledge questions correlated to water policy preferences. Those who correctly answered questions pertaining to the safe use of recycled water to grow food and that irrigation is the largest user of groundwater in the U.S. were much more supportive of conservation policies to address the management of water resources and less supportive of building dams and reservoirs or pipelines. These findings align with the reality that there is no more water to be stored or piped to other water-deprived regions. Therefore, it was expected that those with accurate water knowledge would support policies seeking to conserve existing water for multiple uses and needs. These findings substantiate prior research that found significant correlations between water knowledge and water conservation [70,93]. Further, of the four conservation policies that water knowledgeable people supported, two were incentive-based, suggesting that policies aimed at water conservation should focus on positive incentives to engage the public and garner support for water conservation policy [94].

This study found that water literacy was central to support for water conservation policies, which is an important contribution to the existing academic literature and potentially for future water policy design and implementation. Broadly, this suggests that increasing public water knowledge may be beneficial to gain public support for water conservation policies. As the West continues to struggle with water availability, periods of drought offer poignant opportunities to engage the public with education campaigns about water and could provide a salient policy window to advance water conservation policies. Further, for policymakers, continuing to combine water conservation policy with incentives to large-scale water users to reduce water use through employing water-saving strategies and technologies would mitigate the financial impacts on irrigators and respond to the public’s desire for positive incentives for water conservation. States in the West already utilize these incentives, with Oregon irrigators using the Clean Water State Revolving Fund (CWSRF) loans to improve upon water delivery and efficiency to reduce overall water use [95]. Funding for these programs could potentially increase with a more water-literate public actively supporting water conservation policies.

The lack of water in the West is not a problem that will go away on its own. Active management of water resources is now required to meet current water needs as well as actively planning how to maintain water uses in the future without further exacerbating limited resources. Policies to encourage water efficiency and conservation will require an active engagement with a knowledgeable public and the participation of all water users. This study illustrated how water knowledge leads to greater levels of support for water conservation policies. Policymakers engaged in managing scarce water resources would potentially benefit from continued efforts to increase public water knowledge. Building on existing awareness and concern, particularly in times of drought where impacts are more tangible to the public could prove effective in developing water knowledge and engaging the public in creating more impactful and sustainable water conservation policies, particularly policies that are incentives-based. Beyond large-scale water conservation policies, this could also engage the public in broader water conservation efforts, particularly those who feel that their actions can have a positive impact on water conservation.

While our study provides convincing evidence of the relationship between water knowledge and policy support for supply-side and demand-side water policies, it is important to point out limitations related to the measurement of water knowledge. Water knowledge was assessed based on only two statements. We used the quiz on the food–water–energy nexus developed by Portney et al. [91], which had other questions related to energy and food knowledge. Future research should develop more comprehensive surveys to assess water knowledge among the respondents. Moreover, future studies should employ panel designs to test causal linkages between water knowledge and water conservation policies.

## Figures and Tables

**Table 1:** Survey Response Bias.

State		California		Idaho		Oregon		Washington	
Demographic Variable		Survey Sample	Census Estimates ^2^	Survey Sample	Census Estimates ^2^	Survey Sample	Census Estimates ^2^	Survey Sample	Census Estimates ^2^
Mean Age ^1^		47.7	47.1	52.6	48.0	55.3	49.5	50.3	48.5
Gender ^1^	Male	51.3%	49.5%	50.1%	50%	48.7%	48.4%	48.3%	48.7%
	Female	48.7%	51.5%	49.9%	50%	51.3%	51.6%	51.7%	51.3%
Associates Degree or Higher ^1^		40.3%	36.7%	48.9%	39.1%	38.1%	35.0%	44.8%	38.8%
Median Household Income		USD 50,000–USD 74,999 ^3^	USD 60,883 ^4^	USD 50,000–USD 74,999 ^3^	USD 46,890 ^4^	USD 50,000–USD 74,999 ^3^	USD 49,260 ^4^	USD 50,000–USD 74,999 ^3^	USD 57,224 ^4^

^1^ Among all adults age 18+; ^2^ data obtained from the U.S. 2010 American Community Survey Public Use Microdata Sample; ^3^ survey category 6; ^4^ 2006–2010 adjusted average.

**Table 2:** Independent and Control Variables.

Variable Name	Variable Description	Mean/Standard Deviation
Age	Age in years (range = 18 to 97)	Mean = 51.60 s.d. = 16.83 N = 1796
Gender	Gender dummy variable (1 = female, 0 = male)	Mean = 0.50 N = 1787
Education	Formal educational attainment (1 = less than high school to 8 = postgraduate degree)	Mean = 4.80 s.d. = 1.46 N = 1798
Income	Household income before taxes in 2017 (1 = less than USD 10,000 to 10 = USD 200,000 or more)	Mean = 5.88 s.d. = 1.80 N = 1772
Efficacy	Environmental efficacy index (4 = low efficacy to 20 = high efficacy)	Mean = 14.16 s.d. = 3.94 N = 1793
Climate Change	Climate change beliefs dummy variable (1 = Earth getting warmer because of human activity, 0 = else)	Mean = 0.61 N = 1793
Ideology	Subjective political ideology (1 = very liberal to 9 = very conservative)	Mean = 4.68 s.d. = 1.25 N = 1782

**Table 3:** Public Perception of Recycled Water and Food.

*Statement:* “Recycled Water Cannot Be Safely Used to Grow Food.”					
	California	Idaho	Oregon	Washington	Total
Accurate	12.0%	6.9%	6.1%	4.0%	7.2%
Inaccurate	74.4%	79.8%	83.1%	75.6%	78.3%
Do not know	13.6%	13.3%	10.8%	20.4%	14.5%
N =	433	435	472	446	1786

chi-square = 39.394, *p* = 0.000.

**Table 4:** Public Perception of Groundwater Use.

*Statement:* “Crop Irrigation in the U.S. Uses More Groundwater Than All Other Uses Combined.”					
	California	Idaho	Oregon	Washington	Total
Accurate	24.3%	37.7%	34.3%	31.4%	32.0%
Inaccurate	26.7%	23.1%	12.8%	10.1%	18.0%
Do not know	48.9%	39.1%	52.8%	58.5%	49.9%
N =	423	432	460	436	1751

chi-square = 76.429, *p* = 0.000.

**Table 5:** Logistic Regression Estimates for Water Knowledge ^a^.

		Recycled Water and Food	Crop Irrigation and Groundwater
		Odd Ratio (CI)	Odd Ratio (CI)
Demographics	Age	1.00 (0.99, 1.00)	0.99 ** (0.98, 1.00)
	Gender	0.80 (0.62, 1.02)	0.77 * (0.62, 0.96)
	Education	1.13 * (1.03, 1.24)	1.10 * (1.02, 1.19)
	Income	1.07 (0.99, 1.15)	1.16 *** (1.09, 1.24)
Values	Efficacy	1.12 *** (1.08, 1.17)	1.04 * (1.01, 1.08)
	Climate Change	1.86 *** (1.36, 2.53)	1.42 * (1.07, 1.89)
	Ideology	1.07 (1.00, 1.14)	0.97 (0.91, 1.02)
States	California (Baseline Category)	1	1
	Idaho	1.61 ** (1.14, 2.29)	2.46 *** (1.79, 3.37)
	Oregon	1.74 ** (1.22, 2.48)	1.76 *** (1.29, 2.41)
	Washington	1.04 (0.74, 1.45)	1.44 * (1.05, 1.97)
	N	1712	1679
	Chi-square	158.200 ***	130.299 ***
	Percent correctly classified	80.1%	68.2%
	Cox and Snell R^2^	0.089	0.061
	Nagelkerke R^2^	0.137	0.104

* *p* ≤ 0.05; ** *p* ≤ 0.01; *** *p* ≤ 0.001; ^a^ dependent variables coding: 1 = correct answer, 0 = else.

**Table 6:** Comparison of Policy Support Mean Scores between Respondents with Correct and Incorrect Responses for Recycled Water for Food Question.

*Question:* A Number of Policy Options Have Been Proposed to Manage Water Resources. Please Indicate Your Level of Opposition or Support for Each of the Following. (1 = Strongly Oppose, 2 = Oppose, 3 = Neutral, 4 = Support, 5 = Strongly Support)				
	Policy Options	Recycled Water Is Safe for Food	Recycled Water Is Not Safe for Food and Do Not Know	
		Mean (s.d.)	Mean (s.d.)	T-test
A.	Build dams and reservoirs N = 1782	3.47 (1.18)	3.67 (1.08)	8.54 **
B.	Build pipelines to bring water from other regions N = 1782	3.00 (1.25)	3.48 (1.20)	45.63 ***
C.	Conduct campaigns for voluntary water conservation N = 1782	4.06 (0.98)	3.47 (1.32)	91.83 ***
D.	Give tax incentives for the installation of water- saving equipment N = 1783	4.11 (0.98)	3.57 (1.29)	79.19 ***
E.	Require low water use landscaping N = 1780	3.66 (1.20)	3.40 (1.34)	13.46 ***
F.	Give tax incentives for implementing efficient irrigation systems for agriculture N = 1783	4.11 (0.89)	3.61 (1.22)	82.89 ***

** *p* ≤ 0.01; *** *p* ≤ 0.001.

**Table 7:** Comparison of Policy Support Mean Scores between Respondents with Correct and Incorrect Responses for Groundwater Use Question.

*Question:* A Number of Policy Options Have Been Proposed to Manage Water Resources. Please Indicate Your Level of Opposition or Support for Each of the Following. (1 = Strongly Oppose, 2 = Oppose, 3 = Neutral, 4 = Support, 5 = Strongly Support)				
	Policy Options	Crop Irrigation Uses More Water	Crop Irrigation Does Not Use More Water and Do Not Know	
		Mean (s.d.)	Mean (s.d.)	T-test
A.	Build dams and reservoirs N = 1747	3.28 (1.18)	3.62 (1.13)	32.61 ***
B.	Build pipelines to bring water from other regions N = 1747	3.01 (1.21)	3.16 (1.27)	5.13 *
C.	Conduct campaigns for voluntary water conservation N = 1747	4.19 (0.83)	3.80 (1.18)	48.94 ***
D.	Give tax incentives for the installation of water- saving equipment N = 1748	4.26 (0.81)	3.85 (1.15)	56.20 ***
E.	Require low water use landscaping N = 1745	3.90 (1.14)	3.47 (1.27)	46.01 ***
F.	Give tax incentives for implementing efficient irrigation systems for agriculture N = 1748	4.18 (0.83)	3.92 (1.05)	25.92 ***

* *p* ≤ 0.05; *** *p* ≤ 0.001.

**Table 8:** Logistic Regression Estimates for Water Policies Controlling for Water Knowledge ^a^.

		Supply-Side Approaches		Demand-Side Approaches (Conservation)			
		Policy A	Policy B	Policy C	Policy D	Policy E	Policy F
		Odd Ratio (CI)	Odd Ratio (CI)	Odd Ratio (CI)	Odd Ratio (CI)	Odd Ratio (CI)	Odd Ratio (CI)
Demographics	Age	1.01 *** (1.01, 1.02)	1.00 (0.99, 1.01)	1.01 (1.00, 1.02)	1.00 (0.99, 1.01)	1.01 * (1.00, 1.02)	1.00 (0.99, 1.00)
	Gender	0.74 ** (0.60, 0.91)	0.76 * (0.61, 0.94)	1.33 * (1.00, 1.76)	1.80 *** (1.35, 2.39)	0.62 *** (0.49, 0.78)	2.82 *** (2.09, 3.81)
	Education	1.11 * (1.02, 1.20)	0.98 (0.90, 1.06)	1.12 * (1.01, 1.25)	1.16 ** (1.04, 1.30)	1.08 (0.99, 1.18)	1.11 (0.99, 1.25)
	Income	0.96 (0.90, 1.02)	1.08 * (1.02, 1.16)	1.03 (0.95, 1.12)	1.33 *** (1.22, 1.44)	0.86 *** (0.80, 0.92)	1.29 *** (1.19, 1.41)
Values	Efficacy	0.93 *** (0.90, 0.96)	0.93 *** (0.90, 0.96)	1.33 *** (1.27, 1.39)	1.18 *** (1.13, 1.23)	1.22 *** (1.17, 1.26)	1.13 *** (1.08, 1.18)
	Climate Change	0.74 * (0.56, 0.96)	0.78 (0.59, 1.02)	2.98 *** (2.16, 4.11)	2.41 *** (1.72, 3.36)	1.52 ** (1.15, 2.01)	1.54 * (1.09, 2.17)
	Ideology	1.24 *** (1.17, 1.31)	1.01 (0.95, 1.07)	0.96 (0.89, 1.03)	0.86 *** (0.80, 0.93)	0.80 *** (0.75, 0.85)	0.85 *** (0.79, 0.92)
Knowledge	Recycled Water	1.20 (0.92, 1.58)	0.66 ** (0.51, 0.86)	1.42 * (1.02, 1.97)	1.41 * (1.01, 1.95)	0.67 ** (0.50, 0.90)	2.13 *** (1.53, 2.95)
	Crop Irrigation	0.61 *** (0.48, 0.78)	0.83 (0.66, 1.05)	1.75 *** (1.26, 2.45)	1.70 ** (1.20, 2.42)	1.97 *** (1.52, 2.56)	1.35 (0.95, 1.91)
States	California (Baseline)	1	1	1	1	1	1
	Idaho	0.66 ** (0.48, 0.90)	0.59 *** (0.43, 0.79)	0.95 (0.63, 1.44)	2.45 *** (1.60, 3.74)	1.20 (0.86, 1.68)	3.30 *** (2.15, 5.05)
	Oregon	0.48 *** (0.35, 0.65)	0.28 *** (0.20, 0.38)	1.04 (0.69, 1.56)	1.10 (0.74, 1.65)	1.33 (0.96, 1.85)	1.53 * (1.03, 2.29)
	Washington	0.54 *** (0.40, 0.73)	0.69 * (0.52, 0.92)	1.64 * (1.07, 2.52)	1.38 (0.91, 2.09)	1.56 ** (1.12, 2.18)	1.79 ** (1.19, 2.70)
	N	1673	1673	1673	1674	1671	1674
	Chi-square	281.265 ***	174.330 ***	664.672 ***	519.908 ***	461.931 ***	421.257 ***
	Percent correctly classified	64.0%	65.4%	82.6%	82.5%	74.0%	84.7%
	Cox and Snell R2	0.121	0.079	0.341	0.294	0.205	0.257
	Nagelkerke R^2^	0.206	0.135	0.476	0.409	0.326	0.356

* *p* ≤ 0.05; ** *p* ≤ 0.01; *** *p* ≤ 0.001; ^a^ support and strongly support policy = 1, else = 0.

## Data Availability

Data are available from the P.I. upon successful formal OSU IRB training and following OSU IRB protocols for protection of human subjects.
